# Interpreting scores on multiple sclerosis-specific patient reported outcome measures (the PRIMUS and U-FIS)

**DOI:** 10.1186/1477-7525-8-117

**Published:** 2010-10-11

**Authors:** James Twiss, Lynda C Doward, Stephen P McKenna, Benjamin Eckert

**Affiliations:** 1 Galen Research Ltd, Manchester, UK; 2 Global Health Economics and Outcomes Research, Novartis Pharmaceuticals, Basel, Switzerland

## Abstract

**Background:**

The PRIMUS is a Multiple Sclerosis (MS)-specific suite of outcome measures including assessments of QoL (PRIMUS QoL, scored 0-22) and activity limitations (PRIMUS Activities, scored 0-30). The U-FIS is a measure of fatigue impact (scored 0-66). These measures have been fully validated previously using an MS sample with mixed diagnoses. The aim of the present study was to validate the measures further in a specifically Relapse Remitting MS (RRMS) sample and to provide preliminary evidence of the responder definitions (RD; also known as minimal important difference) for these instruments.

**Methods:**

Data were derived from a multi-country efficacy trial of MS patients with assessments at baseline and 12 months. Baseline data were used to assess the internal reliability and validity of the measures. Both anchor-based and distribution-based approaches were employed for estimating RD. Anchor-based estimates were based on published RD values for the EQ-5D and were assessed for those improving and deteriorating separately. Distribution-based estimates were based on standard error of measurement (SEM), change score equivalent to 0.30, and change score equivalent to 0.50, effect sizes (ES).

**Results:**

The sample included 911 RRMS patients (67.3% female, age mean (SD) 36.2 (8.4) years, duration of MS mean (SD) 4.8 (5.2) years). Results showed that the PRIMUS and U-FIS had good internal consistency. Appropriate correlations were observed with comparator instruments and both measures were able to distinguish between participants based on Expanded Disability Status Scale scores and time since diagnosis. The anchor-based and distribution-based RD estimates were: PRIMUS Activities range = 1.2-2.3, PRIMUS QoL range = 1.0-2.2, and U-FIS range = 2.4-7.0.

**Conclusions:**

The results show that the PRIMUS and U-FIS are valid instruments for use with RRMS patients. The analyses provide preliminary information on how to interpret scores on the scales. These data will be useful for assessing treatment efficacy and for powering clinical studies.

**Trial Reference Number:**

ClinicalTrials.gov Identifier NCT00340834.

## Background

Multiple sclerosis (MS) is a chronic, autoimmune and neurodegenerative disorder of the central nervous system (CNS) characterized by inflammation, demyelination and neuronal loss. MS represents the leading cause of non-traumatic neurologic disability in young and middle-aged adults, affecting an estimated 2.5 million individuals worldwide [1]. About 85% of patients begin with the Relapse Remitting form of MS (RRMS) which is characterised by episodes of symptoms followed by resolution, at least partly, within days to months [2,3]. The long term clinical effects of MS often lead to serious disability. Symptoms of MS are wide ranging and can include weakness of the limbs (particularly the legs), fatigue, unsteadiness, difficulty with bladder control, visual changes due to the involvement of the optic nerve, vertigo, facial numbness or weakness or double vision [4]. In addition, depression occurs in about a quarter of patients [5]. Unsurprisingly, the disease can have major detrimental effects on a patient's QoL [3,6,7].

Measuring the wide ranging effects of MS is important for developing understanding and treatment of this disease. The Patient Reported Indices for Multiple Sclerosis (PRIMUS) was developed to capture the overall impact of MS from the patient's perspective [8]. This instrument consists of three distinct scales specific to MS; symptoms, activity limitations and quality of life (QoL), each designed to be used in combination or as a standalone measure. Scale content was generated directly from MS patients and, consequently closely represents patients' experience of MS. As fatigue is present in about three quarters of patients [9] the Unidimensional Fatigue Impact scale (U-FIS) [10] was developed in parallel with the PRIMUS scales to provide an index of the impact of fatigue associated with MS. The PRIMUS and U-FIS scales were developed and validated in patients representing the most common MS sub-types; RRMS, Secondary Progressive MS and Primary Progressive MS [8,10]. Data from a large 12 month efficacy trial were made available to evaluate the validity of the instruments further specifically for RRMS. These data also provided an opportunity to investigate how to interpret scores for the PRIMUS and U-FIS.

One of the most commonly used approaches for investigating how to interpret scores on Patient Reported Outcome (PRO) scales has been through the calculation of a minimum score that can be considered to be clinically meaningful. This score can then be used to help interpret treatment response during therapeutic trials. Calculation of this score has been referred to as the Minimal Important Difference (MID) [11], meaningful change [12] and minimal clinically significant difference [13]. More recently the term Responder Definition (RD) has replaced previous terminology [14].

No single method for estimating the RD is widely accepted. Approaches can be classified broadly into anchor-based and distribution-based approaches. Anchor-based approaches involve relating change scores on the PRO to change in a factor of known importance. These methods usually involve using other PROs, [11,15,16] clinical variables [17,18] or patient global rating of change questions [12,19,20] as an anchor. Each approach has strengths and limitations. Other comparator instruments can only be used when the instruments are suitably related to the testing instrument and cover issues important and relevant to the patient [21]. Some authors have suggested that a correlation of 0.5 is necessary between the anchor and main instrument in order to ensure adequate relatedness [15,16]. In these cases it is also useful if previous research has investigated the RD of the comparator instrument. Clinical variables can provide useful markers for interpreting scores on PROs but they do not provide minimal important difference estimates per se. These are most useful when other information for estimating RD is unavailable. Global Rating of Change (GRC) questions generally have multiple Likert type response options ranging from 'very much worse' to 'very much better'. Change scores for those individuals responding 'a little' or 'moderately' improved are used to estimate the RD. Although global rating of change questions are easy to administer the reliability of such methods is questionable. Doubt exists about whether patients can recall their health over periods of time and it is unknown whether patients respond primarily in relation to their current health rather than their change in health [22]. It has also been argued that estimation of RD should not be based on GRC items alone [21].

Distribution-based approaches assess the distribution of scores on the PRO and attempt to identify a score that may be considered important above the 'statistical noise' of the measure. Various distribution-based approaches have been suggested including effect size [23], half a standard deviation [24], the standard error of measurement (SEM) [25] and the standard response mean (SRM) [26]. These different approaches usually produce different magnitudes of RD. Furthermore, distribution-based estimates can sometimes differ considerably from those obtained using anchor-based methods [27].

No previous study has attempted to determine the RD of the PRIMUS and U-FIS. The aim of the present study was twofold. First, to provide further evidence of the validity of the PRIMUS and U-FIS in a RRMS sample. Secondly, to investigate the RD of the PRIMUS and U-FIS scales.

## Methods

### Patients

Analyses were based on data collected in a 12-month, randomized, multicenter, double-blind, efficacy trial where patients were randomized to receive a fixed dose of either FTY720 0.5 mg/day orally, FTY720 1.25 mg/day orally or interferon beta-1a 30 μg/week. The trial included 1292 RRMS patients at 172 centers in 18 countries. PRIMUS and U-FIS data were only available for countries where the questionnaires had been previously formally adapted and validated [8,28,10,29]. Data were available for 911 patients from the following 8 countries; Canada (French and English), France, Germany, Italy, Spain, United Kingdom, United States and Australia.

The participants were aged 18 to 55 years, with active MS (defined as one relapse during the previous year or two relapses during the previous 2 years), Expanded Disability Status Scale (EDSS) score of between 0 and 5.5 and neurologically stable for at least 30 days prior to randomization.

### Measures

The PRIMUS consists of three independent scales; symptoms, activity limitations and QoL designed to be used as standalone measures or in combination [8,28]. For the present study data were available for the QoL and activity limitation scales. The QoL scale contains 22-items in the form of simple statements accompanied by dichotomous response options. Items are summed in each scale to yield a total score ranging from 0 to 22. High scores indicate worse QoL. The activity limitations scale contains 15-items describing specific physical tasks. Respondents rate the degree to which they are able to perform the tasks on a three point scale. Again, items are summed to give a total score that can range from 0 to 30. High scores are indicative of greater activity limitation. Both scales have been shown to be unidimensional and to have good reproducibility and validity in a number of languages [28].

The U-FIS has 22-items measuring the impact of fatigue [10,29]. For each item, individuals rate the degree to which they have been affected by fatigue during the previous week on a scale ranging from 'Never' (scored 0) to 'All the time' (scored 3). Item scores are summed to give a total score that can range from 0 to 66. The U-FIS is unidimensional and has been shown to have good reproducibility and validity in several languages [29]. The PRIMUS and U-FIS are available at http://www.galen-research.com.

The Expanded Disability Status Scale (EDSS) is a global scale developed to evaluate disability due to neurologic limitations in people with MS [30]. It has 20 available levels that describe progressive disability ranging from 0 (normal) to 10 (death due to MS) rising in 0.5 units. Patients are clinically assessed and assigned scores in eight functional systems that are scored from 0-5 or 0-6. Higher scores represent greater system impact. The eight functional systems are; pyramidal, cerebellar, brainstem, sensory, bowel and bladder, visual and cerebral/mental functions. EDSS scores are generated from the system functions scores and other information collected during the clinical examination.

The Multiple Sclerosis Functional composite (MSFC) is a clinical measure of physical and cognitive functioning in MS patients [31]. It assesses leg function/ambulation, arm/hand function and cognitive function. These three scales are also added together to give a composite measure of functioning. The leg function/ambulation measure is based on the average of two timed 25-foot walk tests. The arm/hand function measure involves four 9-hole peg tests. The cognitive function measure is the Paced Auditory Serial Addition Test (PASAT) that assesses auditory processing speed and working memory [32]. The three separate scale scores are converted into z-scores before being added together to form a composite score.

The EQ-5D is a generic health outcome assessment [33]. It consists of 5 items: Mobility, Self-care, Usual activities, Pain/Discomfort and Anxiety/depression, each with 3 levels (no problems, moderate problems, extreme problems). A health utility value is derived for each patient based on their combination of responses to the five items. The score is on a continuum from 1 (best possible health) to 0 (death) with some health states being valued worse than death (< 0). Research has suggested that the RD of the EQ-5D is 0.074 [34].

### Statistical analysis

#### Reliability and Validity

The distributional properties of the PRIMUS and U-FIS were explored through descriptive statistics (mean, standard deviation, median and inter-quartile range [IQR]) and floor and ceiling effects (percentage of patients scoring the minimum and maximum possible scores, respectively). Internal consistency (degree of relatedness of items) was assessed using Cronbach's alpha. A correlation of 0.70 is accepted as indicating adequate consistency [35]. Convergent and discriminant validity were evaluated by assessing the level of association (Spearman rank correlations) between scores on the PRIMUS and U-FIS scales and those on the EQ-5D, EDSS and the MSFC subscales and composite score. Known groups validity was assessed by examining the PRIMUS and U-FIS scores of respondents who differed according to their baseline EDSS group and duration of MS. EDSS group was defined in the following way; EDSS (0 - 1.5), EDSS (2 - 2.5), EDSS (3 - 3.5), EDSS (4-5.5). Non-parametric tests for independent samples (Mann-Whitney U Test for two groups and Kruskal-Wallis one-way analysis of variance for three or more groups) were employed. Psychometric testing was performed using the SPSS 17.0 statistical package.

#### Responder Definition Analysis

The RDs for the PRIMUS and U-FIS were estimated using a combination of anchor-based and distribution-based methods. Anchor-based analyses were conducted by comparing scores on the PRIMUS and U-FIS with published RD values for the EQ-5D [34]. The anchor approach assessed change scores for the PRIMUS and U-FIS for individuals who improved or deteriorated by 0.074-0.111 on the EQ-5D (1-1.5 times the RD of the EQ-5D).

The distributional methods included the assessment of effect size, half a standard deviation and standard error of measurement. The effect size (ES) statistic is based on the ratio of difference between a target measure's mean at baseline and at follow-up (related to the standard deviation of the baseline scores). The group change ES is calculated as follows:

$$ES=\frac{\left(m_{2}-m_{1}\right)}{s_{1}}$$

Where m_1 _is the group mean at baseline, m_2 _is the group mean at follow-up and s_1 _is the group standard deviation at baseline. Cohen devised ES thresholds for assessing the magnitude of group change that are widely accepted [23]. These are 0.2 for a small group change, 0.5 for a moderate group change and 0.8 for a large group change. Estimates of change scores needed to produce different effect sizes can be calculated using baseline standard deviations. Half a standard deviation (equivalent to half the baseline standard deviation) is commonly found to be close in value to published RD values [24]. Change scores required to produce effect sizes of 0.3, and 0.5 were calculated.

The SEM has also been posited as a surrogate for the RD [25]. It has been described as the standard error in an observed score that obscures the true score [36]. It is estimated as follows:

$$SEM=s_{1}\times \left(\sqrt{1-r}\right)$$

Standard deviation at baseline (s_1_) is multiplied by the square root of one minus the internal consistency of the target measure (as assessed by Cronbach's Alpha coefficient (r)). SEM has been used frequently to aid in the interpretation of PRO scores and a change above 1 SEM has been considered to be meaningful [37-40].

## Results

Demographic and disease information for the sample is shown in Table 1. The table shows that the sample was relatively mild in terms of MS severity. A majority of patients had EDSS scores between 0 and 2.5 and most reported having had two or fewer relapses in the previous two years.

**Table 1:** Participant details (n = 911)

Sex	
**Male **(%)	292 (32.1)
**Female **(%)	618 (67.8)

**Missing (%)**	1 (0.1)

**Age (years)**	
**Mean (SD)**	36.5 (8.4)
**Median (IQR)**	37 (30 - 43)
**Range**	18 - 55

**Missing (%)**	0

**Duration of MS (years)**	
**Mean (SD)**	4.8 (5.2)
**Median (IQR)**	3.2 (0.7 - 7.2)
**Range**	0.1 - 32.9

**Missing (%)**	9 (1)

**Number (%) relapses in the previous 2 years**	
**1**	268 (29.4)
**2**	536 (58.8)
**3**	86 (9.4)
**4**	18 (2.0)

**Missing (%)**	3 (0.3)

**EDSS Group (%)**	
**0-1.5**	400 (44.3)
**2-2.5**	262 (29.0)
**3-3.5**	135 (15.0)
**4 +**	105 (11.6)

**Missing (%)**	9 (1)

Questionnaire responses on the PRIMUS, U-FIS and EQ-5D are reported in Table 2. Results showed that over 20% of respondents scored the minimum for the PRIMUS Activity limitations and QoL scale and the maximum for the EQ-5D scale (which indicates good health status). These findings confirm the relatively low baseline disability in the sample. Results showed that there were few signs of ceiling effects for the PRIMUS or U-FIS scales.

**Table 2:** Descriptive scores on patient reported outcome measures

	PRIMUS QoL	PRIMUS Activities	UFIS	EQ-5 D Utility
**Baseline**				
N	885	883	873	900
Mean (SD)	4.0 (4.3)	3.0 (4.6)	16.8 (13.9)	0.80 (0.19)
Median (IQR)	2.0 (1.0 - 6.0)	2.0 (0 - 4.0)	14.0 (5.0 - 27.0)	0.80 (0.73 - 1)
% scoring Min	21.4	39.8	7.0	0
% scoring Max	0	0.2	0	29.9

**12 Months**				
n	835	833	825	839
Mean (SD)	3.8 (4.7)	3.2 (4.8)	17.0 (14.8)	0.80 (0.21)
Median (IQR)	2.0 (0 - 6.0)	1.0 (0 - 4.0)	13.0 (4.0 - 27.0)	0.81 (0.73 - 1)
% scoring Min	29.8	41.5	10.4	0
% scoring Max	0.2	0.4	0.2	35.2

### Internal consistency

Cronbach's alpha coefficients for the scales were; PRIMUS Activities 0.88, PRIMUS QoL 0.92, and U-FIS 0.97. As cronbach's alpha coefficients were all above 0.7 this indicated good interrelatedness of items.

### Convergent validity

Correlations between questionnaire and physician assessments are shown in Table 3. As anticipated, moderate correlations were found between the PRIMUS scales/U-FIS and EQ-5D scales as these assess related but distinct constructs. The PRIMUS scales and the U-FIS correlated strongly with each other. The EDSS showed low to moderate correlations with the PRIMUS scales and with the U-FIS. The PRIMUS QoL scale and the U-FIS showed weak associations with the MSFC scales and composite score. The PRIMUS Activities scale showed slightly stronger associations with the MSFC scales and composite but these still remained lower than expected. It should be noted that the EDSS and the EQ-5D also showed lower than expected correlations with the MSFC composite score and its subscales. In particular, all scales correlated weakly with the MSFC PASAT scores.

**Table 3:** Convergent validity PRIMUS QoL, PRIMUS Activities and U-FIS at baseline

	PRIMUS QoL	PRIMUS Activities	U-FIS	Timed 25 foot Walk test	9-hole peg test	PASAT	MSFC Total	EDSS
**PRIMUS Activities**	.62							
**U-FIS**	.75	.66						
**Timed 25 foot Walk test**	.20	.32	.22					
**9-hole peg test**	.20	.31	.22	.31				
**PASAT**	-.17	-.18	-.18	-.20	-.20			
**MSFC Total**	-.24	-.33	-.25	-.47	-.72	.71		
**EDSS**	.35	.65	.38	.27	.34	-.14	-.31	
**EQ-5 D Utility**	-.58	-.58	-.60	-.20	-.23	.14	.24	-.35

All correlations were significant at the <0.01 level (2 tailed, Spearman Rank correlations)

### Known group validity

Results of the known group validity assessments for the PRIMUS and U-FIS sales are shown in Table 4. Each of the scales was able to distinguish between participants based on EDSS group. As expected, individuals with greater disability according to EDSS had significantly higher PRIMUS and U-FIS scores. The PRIMUS scales and U-FIS were also able to distinguish between participants based on their duration of MS. As anticipated, individuals who had experienced MS for longer had significantly higher scores on the scales. The PRIMUS scales and U-FIS were also able to distinguish between individuals based on the number of relapses they had experienced in the previous two years. Significant differences in PRIMUS activity limitations and U-FIS scores were found between groups split by number of relapses in the previous two years. Individuals with more relapses obtained higher scores. There was a similar, but not statistically significant, finding for QoL scores. However, both the PRIMUS QoL and U-FIS scales showed statistically significant differences between patients who reported two relapses compared with those who reported three or more.

**Table 4:** Known Group Validity at baseline

		PRIMUS QoL		PRIMUS Activities		UFIS
	**n**	**Mean (SD)**	**n**	**Mean (SD)**	**n**	**Mean (SD)**

**EDSS Group**						
0-1.5	391	2.7 (3.5)	393	1.6 (3.5)	381	11.7 (11.0)
2-2.5	255	3.8 (4.0)	253	2.7 (3.8)	252	17.6 (13.7)
3-3.5	130	5.3 (4.6)	129	4.5 (5.4)	129	22.2 (14.4)
4-5.5	102	7.4 (5.2)	99	7.7 (5.5)	102	27.1 (14.8)

***P***		< 0.01		< 0.01		< 0.01

**Number of relapses in previous 2 years**						
**1**	259	3.8 (4.3)	260	2.2 (3.4)	262	16.1 (13.8)
**2**	522	3.8 (4.1)	519	3.1 (4.7)	508	16.2 (13.4)
**3+**	101	5.1 (5.3)	101	4.7 (6.3)	100	22.1 (15.4)

***P***		0.084		< 0.01		< 0.01

**Median MS duration group**						
**Below median (3.2)**	439	3.6 (4.2)	435	2.3 (4.1)	435	14.5 (13.3)
**Above median (3.2)**	439	4.3 (4.4)	439	3.8 (5.0)	429	19.1 (14.1)

***P***		< 0.01		< 0.01		< 0.01

Non-parametric tests were conducted (Mann-Whitney U Test for two groups and Kruskal-Wallis one-way analysis of variance for three or more groups)

### Responder definition analysis

The anchor-based estimates for the RD for those improving and deteriorating are shown in Table 5. The results showed that for the PRIMUS Activities and QoL scales the RD estimates were similar for patients who improved or deteriorated. There was a more pronounced difference in RD estimates between patients who improved or deteriorated according to the U-FIS. Note that scores for no change in EQ-5D provided the following change scores; -0.2 (n = 331) for Activity limitations, 0.3 (n = 331) for QoL and 0.0 (n = 325) for U-FIS.

**Table 5:** Responder definition estimates

	PRIMUS Activities		PRIMUS QoL		U-FIS	
**Responder Definition Estimates**	**n**	**Mean** **change** **score**	**n**	**Mean** **change** **score**	**n**	**Mean** **change** **score**

**Anchor-based**						
Equivalent to reported EQ-5 D RD improvement	16	-2.3	17	-1.0	17	-6.5
Equivalent to reported EQ-5 D RD deterioration	15	1.8	15	1.0	15	4.7

**Distribution-based**						
SEM	814	1.2	812	1.5	815	2.4
Change score equivalent to 0.30 ES	814	1.4	812	1.3	815	4.2
Change score equivalent to 0.50 ES	814	2.3	812	2.2	815	7.0

Distribution-based estimates were conducted at baseline

Values for the distribution-based approaches (SEM and ES) are also shown in Table 5. The distribution-based estimates provided similar values to the anchor-based estimates.

The final ranges in RD values for each scale were PRIMUS QoL 1.0-2.2, Activities 1.2-2.3 and U-FIS 2.4-7.0.

## Discussion

The results of this study support the use of the PRIMUS and U-FIS with Relapse Remitting MS samples. Questionnaire descriptive statistics confirmed the mild severity of the sample demonstrated by the clinical data. Internal consistency was above 0.70 for the PRIMUS and U-FIS scales indicating that items in the scales were sufficiently related. Convergent and divergent validity showed that the PRIMUS and U-FIS scales had the expected patterns of association with the comparator measures. Scores on the PRIMUS and U-FIS scales were also related to each other in the same way as was found in previous research involving a wider range of types of MS [8,10]. Associations between the PRIMUS and U-FIS and the MSFC subscales and composite score were weaker than expected. However, associations between the MSFC, EDSS and EQ-5D were also weaker than expected suggesting that further investigation of the relation between the MSFC and other clinical outcome measures is needed [41-44].

Known groups validity results showed that the PRIMUS scales and the U-FIS were able to distinguish between participants based on their EDSS level and duration of illness. The PRIMUS and U-FIS scales were also able to distinguish between participants based on the number of relapses they had experienced in the previous two years, although, this difference was not statistically significant for the PRIMUS QoL scale. However, it may be more appropriate to measure relapse frequency yearly or 6 monthly to provide more accurate information.

The anchor estimates produced preliminary evidence of the RDs for the PRIMUS and U-FIS. Encouragingly, the scores obtained for the anchor-based estimates were similar in value to those obtained from the distribution-based estimates. Previous research has suggested that there may be differences in RD values depending on whether individuals improve or deteriorate [45-47]. In the present study there was no bi-directional difference in anchor-based RD values for individuals who improved or deteriorated for the PRIMUS Activities and QoL scales. However, there was a bi-directional difference for the U-FIS; individuals who improved had an RD of 6.5 compared with 4.7 for those who deteriorated. Despite this difference both the improving and deteriorating anchor values for the U-FIS were within the range of the distribution-based estimates. It is unclear whether there are true differences in the RD values for individuals with improving or deteriorating scores on the U-FIS. Further research is needed to investigate this issue.

The final range in values for each scale can be used to provide preliminary guidance when interpreting changes in scores on the measures and to aid calculation of sample sizes needed for clinical studies. Future research is needed to determine whether the RD estimates remain constant in more severe samples and with different types of MS. Previous researchers have highlighted the possibility that the RD may vary as a function of severity [13,21]. For example, it is possible that individuals with severe forms of Secondary Progressive MS may have higher RDs for the scales. The present study investigated the RDs of the PRIMUS and U-FIS in a fairly mild sample of RM patients and the results can be considered valid for future similar samples.

The study has a number of limitations. As mentioned earlier, the sample included a high proportion of patients at the low end of the MS disability spectrum. However, this is consistent with recent clinical trials of RRMS patients and is likely to be reflected in future RRMS studies where the PRIMUS and UFIS are applied. The present assessments were unable to report on the reproducibility of the PRIMUS and U-FIS scales in this sample. However, previous research, including a large proportion of RRMS patients, indicated that the scales had excellent reproducibility [8,10,28,29]. Anchor-based estimates of RD were based on the published RD value for the EQ-5D. Although this provided a useful tool for the present study there are other potential anchors that could be used such as a global question on change in overall health. Finally, as there was little change in patient condition during the trial, relatively few patients could be included in the RD anchor analysis.

## Conclusions

The PRIMUS and U-FIS have been shown to be reliable and valid instruments for the assessment of outcome in RRMS patients. RD estimates are between 1.2-2.3 for the PRIMUS Activity scale, 1.0-2.2 for the QoL scale and 2.4-7.0 for the U-FIS. These estimates are important to help interpretation of change scores and to assist in determining sample sizes necessary for future clinical studies.

## Abbreviations

MID: minimal clinically significant difference; MS: multiple sclerosis; QoL: quality of life; PRO: patient reported outcome; RD: responder definition; RRMS: Relapse Remitting Multiple Sclerosis.

## Competing interests

The authors declare that they have no competing interests.

## Authors' contributions

JT was involved with the design of the study, analysis and interpretation of data and drafting of the manuscript. LCD was involved in the conception and design of the study, interpretation of data and contributed to the manuscript. SPM was involved with the design of study, interpretation of the data and contributed to the manuscript. BE was involved with the design of the study, acquisition of data and reviewed and contributed to the manuscript. All authors read and approved the final manuscript.
